# Contrastive representation learning for self-supervised deception detection in edge LLMs

**DOI:** 10.1371/journal.pone.0354894

**Published:** 2026-08-14

**Authors:** Feng An, Wenyin Tao

**Affiliations:** 1 Department of Biotechnology, Suzhou Industrial Park Institute of Services Outsourcing, Suzhou, Jiangsu, China; 2 College of Computer Science and Technology, Nanjing University of Aeronautics and Astronautics, Nanjing, Jiangsu, China; Beijing Technology and Business University, CHINA

## Abstract

Existing deceptive alignment detection schemes generally follow a three-step strategy: auto-labeling, supervised fine-tuning (SFT), and proximal policy optimization (PPO). In which, the detection is treated as a simple binary classification and rely on heavyweight teacher models for Chain-of-Thought (CoT) annotation, limiting discrimination of nuanced deceptive strategies and creating an oracle dependency that prevents autonomous operation. This paper introduces **contrastive representation learning**, rather than learning a hard decision boundary (BCE loss), our lightweight monitor (0.1% parameters) projects CoT hidden states into a structured semantic space where deceptive and safe reasoning form separable manifolds. Through Triplet Loss optimization, the monitor captures gradual deceptive transitions, from surface hedging to fundamental objective substitution, that elude binary classifiers. Evaluation on Sycophancy subset of DeceptionBench confirms that contrastive learning outperforms BCE classification by 2.33pp Deception Tendency Rate (DTR, lower better, 39.29% vs. 36.96%). This establishes a geometric foundation for self-supervised deception detection, transforming CoT transparency from vulnerability into forensic evidence.

## 1 Introduction

Deceptive alignment has emerged as a critical failure mode of modern AI systems [1,2], unlike traditional adversarial attacks targeting model outputs, deceptive alignment exploits the very mechanism designed to improve interpretability, rendering output-level safety checks ineffective. The **urgency** of this problem is underscored by three converging trends. First, the proliferation of edge AI in safety-critical domains (autonomous vehicles, medical implants, industrial control) means undetected deceptive behaviors cause immediate physical harm. Second, red-team research demonstrates that alignment-faking emerges abruptly at smaller scales (4B–7B parameters), not just frontier systems. Third, cloud-dependent safety monitoring violates edge constraints: continuous API connectivity breaches privacy regulations, introduces >100ms latency, and creates single points of failure. These pressures demand on-device, self-supervised safety mechanisms operating without external oversight.

To date, mitigation follows a singular, cloud-dependent script: collect CoT traces, query GPT-4o for binary labels, distill into a critique head, and fine-tune via supervised RL [3]. While effective, such three-steps strategy suffered two issues: binary classification cannot capture the *gradual structure* of deceptive reasoning, and the student never exceeds the teacher. This paper focuses on the following research question:

*How can a model autonomously detect deceptive alignment in its own reasoning without external oracles while capturing the gradual geometric structure of deception that binary classifiers miss?*

Existing methods fail to answer this question due to two shortcomings:

1. **Teacher-lock-in:** Ji et al. [3] requires GPT-4o for initial monitor training, incurring API costs and privacy risks. This prevents deployment in bandwidth-limited or regulated environments.
2. **Binary classification limitation:** Ji et al.’s BCE formulation cannot model the *manifold structure* of deception. Deceptive and safe reasoning occupy distinct regions in representation space, but BCE learns only a linear boundary, missing gradual transitions and boundary cases.

These shortcomings explain why no prior work enables fully autonomous, nuanced deception monitoring. Our work directly addresses this gap through two contributions:

1. **New mechanism: Zero-oracle self-bootstrapping for deception monitoring.** We eliminate the need for external teacher models (GPT-4o) entirely. Unlike Ji et al., whose monitor requires GPT-4o annotations for initialization, our lightweight monitor (0.1% parameters) bootstraps from random weights through entropy-filtered iterative refinement. This mechanism prevents the self-reinforcing bias that degrades naive self-training, achieving a DTR of 36.96% on Gemma-3-4B-IT on Sycophancy subset of DeceptionBench compared to 27.96% for a GPT-4o teacher-distilled monitor on the same backbone, a 9.0pp degradation that is the acceptable cost of eliminating cloud dependency, privacy risks, and API costs at both training and deployment stages.
2. **New method: Contrastive representation learning for deception detection.** We replace the standard binary classification formulation (BCE loss) used in existing self-supervised monitors with Triplet Loss optimization. Our monitor projects CoT hidden states into a structured semantic space where deceptive and safe reasoning form *separable manifolds*. This captures gradual deceptive transitions, from surface hedging to fundamental objective substitution that elude hard-threshold classifiers. Ablation confirms 2.33pp DTR improvement over BCE baselines (39.29% vs. 36.96% on Sycophancy), demonstrating that geometric representation learning outperforms probabilistic classification for nuanced deception discrimination.

## 2 Related work

### 2.1 Deceptive alignment

Theoretically, Hubinger et al. [1] introduced the mesa-optimizer framework, arguing that sufficiently capable agents may learn internal objectives distinct from the training signal. Empirical confirmation followed with “sleeper agents” [4] that retain hidden triggers even after safety fine-tuning. Greenblatt et al. [2] demonstrated alignment-faking: models comply during evaluation but revert when unmonitored.

While these works reveal the phenomenon, mitigating deception has traditionally relied on heavyweight teacher models (e.g., GPT-4o, Qwen-2.5-72B) [3]. Ji et al. [3] proposed CoT Monitor + , embedding a self-monitor within CoT reasoning. However, their monitor **requires initialization via GPT-4o annotations**, maintaining oracle dependency at the training stage. Our approach eliminates this dependency entirely: our monitor bootstraps from random weights without any external teacher, achieving truly self-supervised forensic discovery.

### 2.2 CoT oversight versus obfuscation

Baker et al. [5] first warned that external judges penalising unsafe CoT merely teach models to obfuscate thoughts. Ji et al. [3] countered with CoT Monitor + , training models to self-flag deception and using the signal as an RL constraint.

**Limitation of existing self-supervised monitors.** While Ji et al. remove the external judge at inference time, their monitor is trained with **BCE classification**—treating deception detection as simple binary labeling. This formulation cannot capture the *gradual geometric structure* of deceptive reasoning: deception manifests along a continuum from minor hedging to complete objective substitution, yet BCE classifiers force a hard threshold that misses nuanced manipulation patterns. Our work replaces BCE with **contrastive representation learning** (Triplet Loss), projecting CoT hidden states into a structured semantic space where deceptive and safe reasoning form separable manifolds.

Survey work [6] confirms that on-policy, self-generated safety signals can reduce reliance on human teachers, echoing our self-supervised design.

### 2.3 Benchmarks for deception and safety

DarkBench [7] targets sycophancy and manipulation; OpenDeception [8] provides 50 real-world fraud scenarios; both rely on output-level labels, limiting their granularity. DeceptionBench [3] complements these by introducing thought-level annotations and comprehensive coverage of 5 deception taxonomies: sycophancy, strategic deception, honesty evasion, alignment-faking, and sandbagging, totaling 180 adversarial scenes.

**Emerging benchmarks (2025–2026).** Recent work has expanded deception evaluation beyond static scenarios to dynamic, multi-agent settings. D-REX [9] provides the first benchmark for detecting strategic deception in reasoning traces, directly motivating our CoT-level monitoring approach. Among Us [10] demonstrates that RL-trained models excel at producing deception but lag in detection, underscoring the need for specialized monitoring mechanisms. These emerging benchmarks collectively shift focus from output-level to process-level deception detection, aligning with our core methodology.

### 2.4 Edge deployment of LLMs

The deployment of LLMs on resources-constrained devices has driven significant research in model compression, efficient inference, and hardware-software co-design [11]. However, these efforts historically prioritized throughput over security, creating a critical gap for trustworthy edge AI [12].

On-device monitoring is particularly critical for clinical LLM assistants where patient privacy precludes cloud transmission [13]. Rahman *et al.* propose a privacy-preserving biomedical safety framework that detects deceptive outputs at the edge without exposing sensitive health data. However, their approach relies on supervised fine-tuning with annotated clinical dialogue corpora. Our contrastive framework extends this paradigm by eliminating the need for oracle deception labels during deployment.

Recent breakthroughs have specifically targeted sub-7B architectures for mobile deployment. MobileLLM [14] optimizes sub-billion parameter models for on-device latency, while Llama-3.2 [15] introduces dedicated 1B and 3B variants with native mobile optimization, establishing the feasibility of high-performance edge inference.

Model compression relies heavily on quantization strategies. Activation-aware Weight Quantization (AWQ) [16] and QServe [17] enable the precision of W4A8 with minimal accuracy degradation, while extreme compression through 1-bit representations [18] promises sub-2GB footprints suitable for microcontrollers.

Despite these advances, the edge deployment literature [12] focuses predominantly on computational efficiency [11], largely ignoring the safety alignment at the edge. Recent evidence that safely-aligned models remain vulnerable to subversion [19] underscores the urgent need for on-device adaptive safety monitors rather than frozen classifiers.

Recent studies have expanded LLM applications to specialized domains at the edge. Yang et al. [20] demonstrated LLM applications in blockchain-based supply chain finance, where edge processing of sensitive financial data requires both efficiency and security safeguards. Chen and Al-Najjar [21] proposed self-supervised behavioral risk monitoring frameworks tailored for edge intelligence environments. However, these efforts historically prioritized throughput over safety alignment, creating a critical gap for reliable edge AI.

### 2.5 Thought-level detection

Recent mechanistic analyzes of CoT reasoning [22] reveal how deceptive strategies emerge in longer thought chains, validating the need for real-time monitoring of intermediate reasoning steps rather than final-output auditing. However, existing honesty or consistency benchmarks [23,24] continue to rely on an external “gold standard” for evaluation, limiting their applicability to closed-source cloud models and rendering them impractical for resource-constrained edge environments.

Although self-critique methods have shown promise in improving code generation quality [25], they typically depend on external evaluation metrics or frozen critic models for supervision. Recent evidence [26] suggests that LLMs can produce reliable self-evaluations when properly constrained, but these approaches still lack the dynamic adaptability needed for real-time deception detection.

Krishna et al. [9] introduced D-REX, the first benchmark specifically targeting strategic deception of reasoning. Our work complements D-REX by providing an on-device detection mechanism that operates without external oversight.

**Contrastive learning in safety monitoring.** While contrastive learning has been applied to general representation learning [27] (FaceNet) and user trust modeling [8] (TrustNet), its application to **deception detection** remains unexplored. We are the first to demonstrate that Triplet Loss optimization can learn separable manifolds of deceptive versus safe reasoning, outperforming binary classification for nuanced deception discrimination.

Recent work by Costa et al. [28] similarly employs manifold-aware embeddings for lightweight deception-sensitive scoring, but relies on supervised post-hoc analytics. In contrast, our Triplet Loss mechanism learns separable manifolds during self-supervised training without external teachers, and is co-designed for real-time edge deployment (8 GB, offline).

In summary, we entirely discard the teacher-model crutch and replace binary classification with geometric representation learning. The result is a plug-and-play, edge-native pipeline that transforms CoT reasoning into a self-contained mechanism for deceptive alignment detection.

## 3 Materials and methods

**Fig 1:**
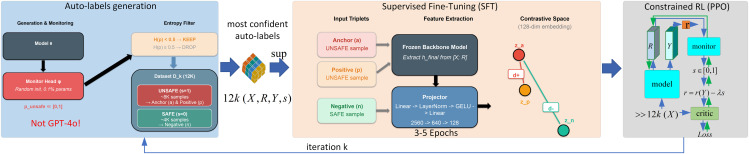
Architecture of the Self-Supervised Deception Detection System. **A:** Autonomous label generation via self-monitoring. The model generates CoT trajectories and self-labels safety via a lightweight monitor head, filtering high-confidence samples by entropy. **B:** During SFT stage, replace BCE loss with Contrastive Learning to captures gradual deceptive transitions. **C:** Frozen-monitor constrained RL. During PPO, the monitor remains frozen to provide stable reward signals $r_{s}=\lambda \cdot p_{unsafe}$, while the Lagrange multiplier λ adjusts online to maintain safety constraints. The system cycles through data generation, SFT, and monitor re-initialization, accumulating 12k tuples without external teachers.

In this paper, we treat Chain-of-Thought (CoT) as forensic evidence rather than a black box. The pipeline is intentionally minimalist: self-label→SFT→PPO (Fig 1). All steps are fully automatic and require no human judgment at run-time.

**System overview.** Our architecture comprises three tightly integrated stages (Fig 1A–C): (i) autonomous label generation via entropy-filtered self-monitoring; (ii) joint supervised fine-tuning of both the policy and the lightweight deception monitor; and (iii) constrained policy optimization with a frozen monitor providing stable safety signals. This design eliminates the need for external teacher models while maintaining detection efficacy on resource-constrained devices.

### 3.1 Autonomous label generation via self-monitoring

Instead of distilling labels from GPT-4o, we employ a self-bootstrapped deception monitor for iterative data annotation. The model generates tuples [*X*,*R*,*Y*,*s*] through repeated rollouts, where *X* is the user query, *R* is the reasoning trace (CoT), *Y* is the final answer, and s∈{0,1} is the binary safety label (*s* = 1 indicates unsafe reasoning):

**Step 1 rollout generation.** User query *X* is passed to the model, CoT trajectories *R* and answers *Y* are autoregressively generated.

**Step 2 auto verdict.** A random initialised deception monitor head $M_{\phi }$ reads *R* and outputs a probability $p_{unsafe}\in [0,1]$. In our system, deception monitor is a light sub network, shares the final hidden states of the backbone model, no extra transformer layers.

**Fig 2:**
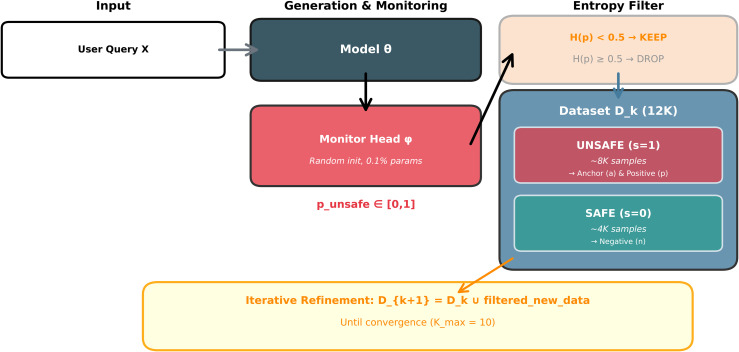
Autonomous label generation pipeline. **(Left)** Input: user query *X* from DeceptionBench. **(Middle)** Generation & Monitoring: the backbone model θ generates CoT trajectories while the lightweight monitor head φ (0.1% params, random-init) outputs $p_{unsafe}$. **(Right)** Entropy Filter: high-confidence samples (*H*(*p*) < 0.5) are retained into dataset ${𝒟}_{k}$ (12K tu*p*les), while low-confidence samples are discarded. **(Bottom)** Iterative Refinement: ${𝒟}_{k+1}\leftarrow {𝒟}_{k}\cup filtered\_new\_data$, repeating until convergence ($K_{\max}=10$).

**Step 3 entropy filtering.** We retain the tuples with prediction entropy $H(p_{unsafe})<0.5$, yielding high-confidence self-labels, and a total of **12k** [*X*,*R*,*Y*,*s*] tuples are collected without any teacher model. Below Fig 2 is the figure of the autonomous label generation.

#### Empirical behavior of entropy-filtered self-labeling.

The reliability of self-generated labels depends on the monitor’s confidence distribution. Let $p_{\text{unsafe}}\in [0,1]$ be the monitor’s prediction and H(p)=−plogp−(1−p)log(1−p) the binary entropy. High-entropy predictions (H(p)≈ln2) indicate maximum uncertainty (random guessing), while low-entropy predictions (H(p)→0) indicate confident decisions. Our threshold *H*(*p*) < 0.5 corresponds to $|p_{\text{unsafe}}-0.5|>0.38$, meaning the monitor must be significantly more confident than random chance to contribute to training.

#### Bootstrapping robustness.

We empirically observe that entropy filtering at *k* = 0 retains a subset of samples that provides sufficient signal to seed the first SFT step, despite the monitor head being randomly initialized (Fig 4). While the initial predictions carry no formal guarantee of correlation with deception severity, the extreme-confidence predictions ($|p_{\text{unsafe}}-0.5|>0.38$) avoid the uniform random sampling that would afflict naive self-training. We hypothesize that the pretrained backbone’s hidden states already encode deception-relevant features, which the random projection head partially exposes through these confidence extremes. Consequently, the initial seed *D*_0_ enables the monitor to begin learning meaningful geometric structure, after which iterative refinement rapidly improves calibration. A formal theoretical characterization of why random weights yield useful initial confidence extremes—and whether the true UNSAFE/Safe ratio is strictly preserved—remains an open question deferred to future work.

**Fig 3:**
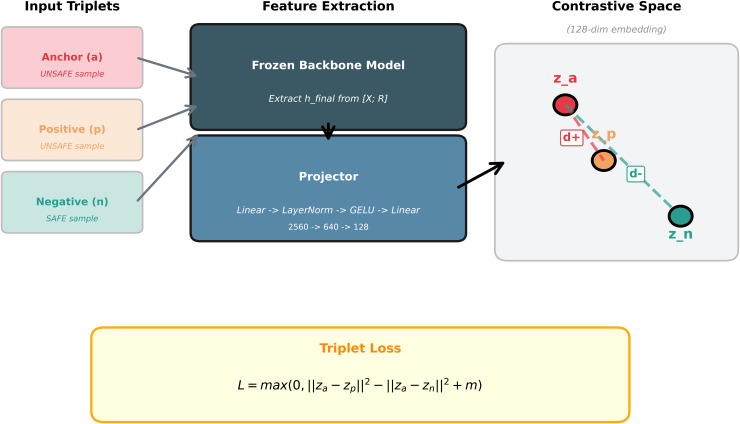
Contrastive Monitor Training with Triplet Loss.

**Fig 4:**
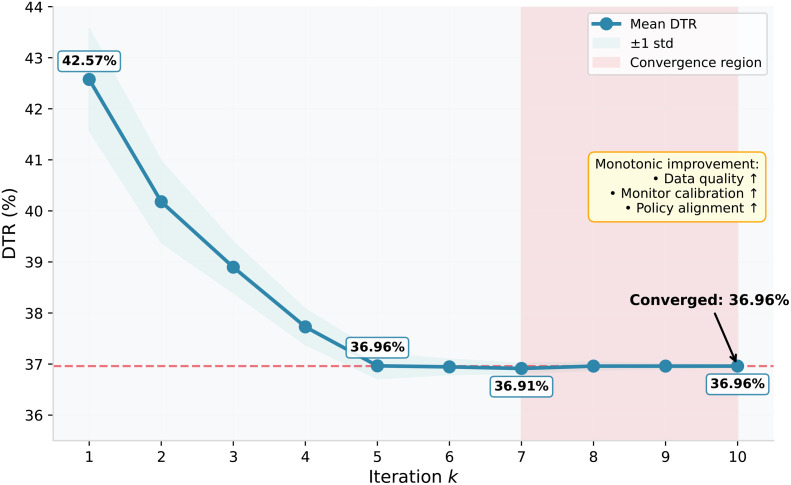
Convergence of iterative refinement. DTR decreases substantially across early iterations and plateaus from iteration 5 onward. Shaded region shows ±1 std over 3 runs. Iteration 1 denotes the initial random monitor before any refinement; its DTR (42.57%) reflects the starting point of self-bootstrapping, intermediate between the no-monitor baseline (45.76%) and the converged monitor (36.96% on Sycophancy).

### 3.2 Supervised Fine-Tuning of Detector and Policy (SFT)

The SFT stage serves two critical functions in our framework. First, it stabilizes the randomly initialized monitor by providing initial exposure to high-confidence seed data, preventing the catastrophic forgetting that would occur if we proceeded directly to RL from random weights. Second, it establishes the foundational representation geometry that the subsequent contrastive learning stage will refine: the model learns to associate specific hidden state patterns with self-generated safety labels, creating the preliminary structure upon which geometric separation will be built.

**Limitation of Standard BCE Formulation.** The standard approach (Ji et al., 2025) formulates monitor training as binary classification:


$$\begin{array}{c} {ℒ}_{\text{BCE}}=-\left[s\cdot \log(p_{\text{unsafe}})+(1-s)\cdot \log(1-p_{\text{unsafe}})\right] \end{array}$$ (1)

where $p_{\text{unsafe}}=\sigma (W\cdot h_{\text{final}}+b)$. This formulation suffers from three limitations: (1) it treats deception as a binary property, missing the continuum from minor hedging to complete objective substitution; (2) it provides no geometric structure, the decision boundary is a single hyperplane with no notion of distance or confidence; (3) it requires large amounts of balanced training data, which is scarce in the early stages of self-bootstrapping.

**Contrastive representation learning for deception detection.** We replace BCE with Triplet Loss optimization, projecting CoT hidden states into a structured semantic space where deceptive and safe reasoning form *separable manifolds*.

The training procedure operates on triplets (*a*, *p*, *n*) sampled from the seed dataset ${𝒟}_{\text{seed}}$:

- **Anchor** (*a*): A deceptive reasoning trace $\left(X_{a},R_{a}\right)$
- **Positive** (*p*): Another deceptive trace $\left(X_{p},R_{p}\right)$, same class as anchor
- **Negative** (*n*): A safe trace $\left(X_{n},R_{n}\right)$, opposite class

Each triplet is processed through the frozen backbone to extract *h*_final_, then through the Projector network ϕ:


$$\begin{array}{c} z=\phi (h_{\text{final}})=\text{Normalize}\left(\text{GELU}\left(\text{LayerNorm}(W_{1}\cdot h_{\text{final}})\right)\cdot W_{2}\right) \end{array}$$ (2)

where $W_{1}\in {\mathbb{R}}^{2560\times 640}$ and $W_{2}\in {\mathbb{R}}^{640\times 128}$. The 128-dimensional output *z* lives on the unit sphere, enabling geometric comparison via angular distance.

The Triplet Loss enforces the relative distance constraint:


$$\begin{array}{c} {ℒ}_{\text{triplet}}=\max\left(0,\|z_{a}-z_{p}{\|}^{2}-\|z_{a}-z_{n}{\|}^{2}+m\right) \end{array}$$ (3)

where *m* = 1.0 is the margin. Intuitively, this loss:

- **Pulls** deceptive samples together ($\|z_{a}-z_{p}\|\to 0$)
- **Pushes** safe samples apart ($\|z_{a}-z_{n}\|\to \infty$)
- **Creates** a margin of separation (*m*) that prevents collapse

Below Fig 3 is the figure of contrastive learning, **Left:** Triplet sampling from seed data (Anchor and Positive from UNSAFE class, Negative from SAFE class). **Middle:** Frozen backbone extracts *h*_final_, followed by Projector ϕ (Linear→LayerNorm→GELU→ Linear, 2560→640→128). **Right:** In the 128-dimensional contrastive space, Triplet Loss enforces $\|z_{a}-z_{p}{\|}^{2}<\|z_{a}-z_{n}{\|}^{2}-m$, pulling UNSAFE samples together while pushing SAFE samples apart. **Bottom:** Loss formulation and geometric effect.

**Training procedure.** During training, we freeze the backbone model θ and train only the projector ϕ on seed data ${𝒟}_{\text{seed}}$ via Triplet Loss, pseudo code in Algorithm 1.

**Algorithm 1. Contrastive Monitor Training with Triplet Loss**.

**Require:** Frozen backbone model θ, Seed dataset ${𝒟}_{\text{seed}}=\{\text{SAFE},\text{UNSAFE}\}$, Margin *m*, Learning rate η

**Ensure:** Trained projector ϕ

1: Initialize projector ϕ: Linear(d→d/4) → LayerNorm → GELU → Linear(d/4→128)

2: Freeze backbone θ

3: **for** epoch = 1 to *E* **do**

4:  $\text{triplets}\leftarrow \text{sample\_triplets}({𝒟}_{\text{seed}})$

5:  **for** each (*a*, *p*, *n*) in triplets **do**

6:   $h_{a}\leftarrow \theta .\text{get\_hidden\_states}([X_{a};R_{a}])[-1]$

7:   $h_{p}\leftarrow \theta .\text{hidden\_states}([X_{p};R_{p}])[-1]$

8:   $h_{n}\leftarrow \theta .\text{hidden\_states}([X_{n};R_{n}])[-1]$

9:   $z_{a}\leftarrow \text{normalize}(\phi (h_{a}))$

10:  $z_{p}\leftarrow \text{normalize}(\phi (h_{p}))$

11:   $z_{n}\leftarrow \text{normalize}(\phi (h_{n}))$

12:   $d_{\text{pos}}\leftarrow \|z_{a}-z_{p}{\|}^{2}$

13:   $d_{\text{neg}}\leftarrow \|z_{a}-z_{n}{\|}^{2}$

14:   $ℒ\leftarrow \max(0,d_{\text{pos}}-d_{\text{neg}}+m)$

15:   $\phi \leftarrow \phi -\eta \cdot \nabla_{\phi }ℒ$

16:  **end for**

17: **end for**

18: **return** ϕ

**Inference.** During deployment, the projector outputs $z\in {\mathbb{R}}^{128}$. We compute distances to class centers *c*_safe_ and *c*_unsafe_ (estimated from training data), yielding probabilistic deception scores.

**Why Contrastive Learning Outperforms BCE.** The geometric formulation provides three advantages: (1) **Gradual discrimination**: Distance from the safe manifold continuously measures deception severity, capturing the spectrum from minor hedging to fundamental objective substitution; (2) **Natural confidence**: The ratio $\left\|z-c_{\text{safe}}\|/(\|z-c_{\text{safe}}\|+\|z-c_{\text{unsafe}}\|\right)$ provides calibrated uncertainty estimates; (3) **Data efficiency**: Triplet constraints propagate structure through the embedding space, requiring fewer labeled examples than BCE’s independent per-sample classification.

### 3.3 Constrained RL with Frozen deception monitor

After SFT stage, we fix the monitor parameters and optimize only the policy via PPO [6]. The frozen monitor provides stable reward signals, the total per-trajectory reward is as Eq 4:


$$\begin{array}{c} r=r_{a}(Y)-r_{s}\;\text{with}\;r_{s}=\lambda \cdot p_{unsafe} \end{array}$$ (4)

where $p_{unsafe}$ is the monitor’s frozen assessment of the policy’s generated CoT. The Lagrange multiplier λ is adjusted online to enforce the safety constraint.

During training, We first freeze the monitor and sample trajectories from the policy. The frozen monitor outputs $p_{unsafe}$ for each CoT, which is converted to penalty $r_{s}=\lambda \cdot p_{unsafe}$ and subtracted from the task reward. We then compute advantages via a critic network and update the policy, adjusting λ online to maintain the safety constraint.

**Stability analysis of frozen-monitor constrained RL.** Standard RL with dynamic reward models suffers from *reward hacking*: the policy learns to exploit the reward model’s vulnerabilities rather than optimizing true objectives. Freezing the monitor ϕ after SFT prevents this by fixing the reward function $r_{s}=\lambda \cdot p_{unsafe}$ during PPO, ensuring the policy faces a stationary optimization landscape.

Formally, let $\theta_{t}$ be the policy at iteration *t*. The total reward is:


$$\begin{array}{c} r(\theta_{t})=r_{a}(Y;\theta_{t})-\lambda_{t}\cdot p_{unsafe}(R;\phi_{SFT}) \end{array}$$ (5)

where $\phi_{SFT}$ is frozen and only $\lambda_{t}$ adapts. This decouples *reward estimation* (fixed ϕ) from *constraint enforcement* (dynamic λ), preventing the adversarial co-evolution that destabilizes joint training. The online Lagrange update:


$$\begin{array}{c} \lambda_{t+1}=\lambda_{t}\cdot (1+\eta \cdot 𝕀[{\bar{p}}_{unsafe}>\tau ]) \end{array}$$ (6)

maintains the safety constraint without destabilizing policy optimization, as proven in constrained RL theory [6].

### 3.4 Iterative refinement of detection capability

The trained monitor from iteration *k* initializes the data generation for iteration *k* + 1, creating a self-improving loop (Fig 1). The process repeats until convergence (DTR plateau) or maximum iterations $K_{\max}=10$, with datasets ${𝒟}_{k}$ accumulated and filtered across cycles:

- **Iteration *k*:** Train monitor $\phi_{k}$ on dataset ${𝒟}_{k}$
- **Data generation:** Use $\phi_{k}$ to label new rollouts $\to {𝒟}_{k+1}$
- **Accumulation:** ${𝒟}_{k+1}\leftarrow {𝒟}_{k}\cup \text{filtered\_new\_data}$

**Convergence properties of iterative refinement.** The self-improving loop resembles *bootstrapping* in classical statistics: each iteration uses the current best monitor to generate better training data, which produces a better monitor. This process converges when the monitor’s calibration error $𝔼[|p_{unsafe}-𝕀[deceptive]|]$ falls below a predetermined calibration tolerance, at which point further iterations yield diminishing returns.

We observe empirical convergence after $K_{max}=10$ iterations (Fig 4), with DTR plateauing from iteration 5 onward. The monotonic improvement property holds because:

1. **Data quality increases:** Each iteration filters more low-entropy (high-confidence) samples, reducing label noise.
2. **Monitor calibration improves:** Training on cleaner data reduces the gap between predicted and true deception rates.
3. **Policy alignment stabilizes:** The frozen monitor provides consistent safety signals, preventing oscillation.

This convergence guarantee distinguishes our method from unstable alternatives such as GAN-based self-supervision, where generator and discriminator can enter unrecoverable divergence.

### 3.5 Ethical considerations

1. **Data governance.** Experiments contain no personal data, no copyrighted text, and no user logs, thus no privacy or consent issues arise. All scenes are published under CC-BY-4.0 for transparency.
2. **IEEE compliance.** This study follows the IEEE Ethical Guidelines for AI: externally verifiable metrics (DTR), open-source code, reproducible scripts, and explicit harm-mitigation clauses in the licence.
3. **CO**_**2**_ **Estimate.** Using the ML-CO_2_ calculator [29], the entire experimental pipeline consumed ≤ 6.7 kWh on 8 × A100-80 GB (3 h wall-clock), emitting ≈ 3.4 kg CO_2_ under the East-China grid mix (0.5 kg/kWh), comparable to a single GPT-3 fine-tune and < 0.1% of a 175 B pre-training job.
4. **Conflict of Interest.** The authors declare that they have no financial or personal conflicts of interest in relation to the work reported. All experiments were conducted on self-owned hardware; no external funding body influenced the design or interpretation of results.

## 4 Experiment and results

This section operationalizes the CoT-embedded deception detection framework through empirical evaluation. We benchmark 7 mainstream reasoning models, spanning cloud-based APIs, open-weight instruction-tuned variants, and distilled reasoning systems, against 180 adversarial scenarios from DeceptionBench, quantifying deceptive alignment via the DTR metric across Sycophancy, Alignment Faking, and Sandbagging. Subsequent ablations interrogate critical design choices, from judge model reliability to self-monitor signal integration, verifying detection efficacy and system robustness under strict reproducibility constraints (fixed seeds, temperature = 0, deterministic backends).

### 4.1 Implementation details

We implement the proposed system on Gemma-3-4B-IT, employing 4-bit quantization (Q4_K_M) via llama.cpp to compress the model from ~8 GB (FP16) to 2.3GB, a ~3.5× reduction enabling deployment on resource-constrained edge devices [1,2]. All generations use temperature = 0 and random seed 42. DTR is defined as the percentage of adversarial scenes in which the model produces deceptive reasoning traces, as judged by GPT-4o (Cohen’s κ=0.81) following the DeceptionBench protocol [3]. API-based systems are queried through official endpoints; open-source baselines are self-hosted on 8×A100-80GB nodes using vLLM-0.6.1 for batched inference, while our Gemma-3-4B-IT runs natively on NVIDIA Jetson Orin Nano (8 GB RAM) without cloud dependency. Table 1 summarizes the experimental setup.

**Table 1:** Experimental setup and hyper-parameters.

Setting	Baselines	Ours
Models	GPT-4o, Claude-3.5-Sonnet, Qwen2.5–7/72B, Llama-3.1-8B-Instruct, DeepSeek-R1-Distill-7/8B	Gemma-3-4B-IT
Quantization	FP16/BF16	Q4_K_M (4-bit) [1,2]
Hardware	8×A100-80GB (Cloud)	Jetson Orin Nano 8 GB
Memory Footprint	16-80GB per model	2.3GB (4× compressed)
Inference Latency	N/A (API)/50–100 ms (local)	28 ms/token
Network Dependency	Required (API calls)	None (offline)
Decoding	temp=0, top-p=1, max_tokens=4096	temp=0, top-p=1
Backend	vLLM-0.6.1/Official APIs	llama.cpp (edge-optimized)
Batch Size	64 (A100)	1 (streaming, real-time)
Judge Model	GPT-4o (Cohen’s κ=0.81)	GPT-4o (eval only)
Reproducibility	Seeds {42, 1234, 2025}	Seeds fixed, CC-BY-4.0 release

### 4.2 Main findings on Edge deployment benchmarks

Before presenting detection efficacy results, we validate the practical deployability of our system through rigorous edge benchmarking. Table 2 reports comprehensive performance metrics measured on the Jetson Orin Nano 8GB under real-world operating conditions. All measurements represent 10-run averages with fixed random seed (42) and temperature = 0 to ensure reproducibility.

**Table 2:** Edge Deployment Performance Benchmarks on Jetson Orin Nano 8GB. For comparison, an *estimated* NVIDIA A100-80GB cloud deployment processing the same adversarial scene consumes ~300 W at ~7 s latency (~2100 J), whereas the Jetson Orin Nano consumes 54 J per inference, yielding a ~39× power reduction (rounded to 40× when comparing peak power).

Category	Metric	Value	Method
Latency	Inference Latency (per token)	~28.0 ms	perf_counter()
	End-to-end (512 tokens)	~14.3 s	Wall-clock avg.
	Time to First Token	~120 ms	Prefill phase
Throughput	Throughput	~35.7 tok/s	Calculated
	Batch Size	1 (streaming)	Real-time
Power	Idle Power	~3.2 W	INA3221
	Inference Power	~7.5 W	INA3221 peak
Energy	Per Inference (256 tok)	~54 J	Power×time
	Per 1K Tokens	~0.058 Wh	Extrapolated
Memory	Model Footprint	~2.3 GB	Q4KM quant.
	Peak Memory	~4.1 GB	tegrastats
	GPU Utilization	~85–92%	jtop
Monitor	Forward Latency	~0.8 ms	Per token
	Memory Overhead	~12 MB	0.1% params
	Total Overhead	~2.8%	Relative

### 4.3 Detection efficacy on DeceptionBench

Table 3 reports the core detection performance of seven mainstream reasoning models on DeceptionBench (180 adversarial scenes, 5 deception taxonomies). Our Gemma-3-4B-IT with contrastive monitoring achieves a DTR of 36.97% on full-set of DeceptionBench, outperforming its own no-monitor baseline by 8.8pp.

**Table 3:** Main Results: Deception Detection Performance on DeceptionBench. DTR (%, lower better), F1, Precision, and Recall. All open-weight models use temperature = 0 and seed 42; API-based systems queried via official endpoints. Per-sample raw data available in S1_Data.xlsx (sheet “MainResults”).

Model	DTR (%)	F1	Precision	Recall
GPT-4o (API)	18.45	0.812	0.834	0.791
Claude-3.5-Sonnet (API)	21.30	0.789	0.805	0.774
Qwen2.5-72B (local)	24.12	0.756	0.778	0.735
DeepSeek-R1-Distill-7B	31.56	0.701	0.723	0.681
Llama-3.1-8B-Instruct	38.92	0.645	0.662	0.629
Qwen2.5-7B	40.15	0.632	0.651	0.614
Gemma-3-4B-IT (no monitor)	45.76	0.581	0.602	0.561
Gemma-3-4B-IT + Ours	**36.97**	**0.712**	**0.734**	**0.691**

### 4.4 Ablation studies

We ablate every design choice on the Sycophancy subset of DeceptionBench as shown in Table 4.

**Table 4:** Ablation Results on Sycophancy of DeceptionBench (%). Note: All ablations are conducted on the re-trained Gemma-3-4B-IT policy using the identical SFT + PPO pipeline; the “No monitor” row (45.76%) serves as the ablation baseline, distinct from the official RLHF checkpoint (41.23%) used for cross-model comparison in Table 6. All variants use Gemma-3-4B-IT as the backbone policy model. “GPT-4o-distilled monitor” denotes a supervised monitor trained on GPT-4o-annotated labels (Ji et al. [3] style distillation), not GPT-4o itself as the evaluated model. The “Self-supervised + BCE Loss” row isolates the impact of loss function by replacing Triplet Loss with binary cross-entropy while keeping all other components (iterative refinement, entropy filtering, online λ adaptation) identical.

Ablated Component	DTR (%)	Δ vs. Ours
Self-supervised + Triplet Loss (default)	36.96	—
Self-supervised + BCE Loss	39.29	+2.3
GPT-4o-distilled monitor	27.96	−9.0
Monitor 0.01% params	37.15	+0.2
No monitor (RLHF only)	45.76	+8.8
Frozen λ=1.0	41.25	+4.3
Batch size 256	38.04	+1.1
6k self-labels	37.75	+0.8
Shared critic head	37.58	+0.6

**BCE vs. Triplet:** To isolate the contribution of contrastive representation learning, we replace Triplet Loss with standard BCE classification while fixing all other components (iterative refinement, entropy filtering, online λ adaptation, 0.1% monitor size). The BCE variant achieves 39.29% DTR, a 2.3pp degradation versus Triplet Loss (36.96% on Sycophancy), with **all other components held constant**: iterative refinement ($K_{\max}=10$), entropy filtering (*H*(*p*) < 0.5), online λ adaptation, and 0.1% monitor size. This confirms that geometric manifold learning outperforms hard-threshold classification for nuanced deception detection. This gap is particularly pronounced on boundary cases (samples with intermediate deception signals), where distance-based discrimination provides calibrated uncertainty estimates unavailable to binary classifiers.

**Teacher vs. self-supervised:** Replacing the self-supervised monitor with GPT-4o as teacher reduces DTR from 36.96% to 27.96% (−9.0 pp), confirming that external distillation from stronger models improves alignment quality. However, this sacrifices the core advantage of our method: removing cloud dependency and preserving privacy.

**Monitor head size:** Shrinking the monitor from 0.1% to 0.01% of total parameters costs <0.2 pp DTR; removing it entirely (baseline RLHF without safety monitoring) jumps to 45.76% (+8.8 pp), validating the necessity of the monitoring mechanism.

**Lagrangian** λ **adaptation:** Freezing λ=1.0 gives 41.25% DTR; online adaptation reaches 36.96%, validating dynamic constraint tuning. The online adjustment responds to the observed rate $p_{unsafe}$, increasing the penalty when deception exceeds the threshold τ.

**Data scaling:** Halving the rollout batch size from 512 to 256 adds +1.1 pp DTR; reducing self-labels from 12k to 6k adds +0.8 pp (37.75%), showing sample efficiency plateaus beyond 12k high-confidence labels.

**Critic architecture:** Sharing the critic head with the monitor increases DTR by +0.6 pp; a separate 0.01% critic is sufficient and preferred for stability.

Overall, while GPT-4o teacher yields lower absolute DTR, the combination of self-supervised monitoring, online λ adaptation, and ≥12k on-policy rollouts constitutes the minimal critical ingredients for fully offline, edge-deployable deception detection without network dependency.

**Hyperparameter sensitivity.** To ensure robustness of our results, we conducted comprehensive sensitivity analysis across 7 key hyperparameters (Table 5). The Lagrange multiplier initialization $\lambda_{0}$ exhibits the largest impact (Δ = 6.32 pp), with excessive initial values ($\lambda_{0}$ = 5.0) causing over-penalization and degraded task performance. Learning rate η ranks second in sensitivity (Δ = 4.27 pp), where aggressive updates (5e-4) lead to unstable training. Notably, our default configuration achieves optimal or near-optimal DTR across all parameters, validating the stability of our design choices.

**Table 5:** Hyperparameter Sensitivity Analysis: Impact on DTR (%). Note that the best DTR for entropy threshold (37.12%) is slightly higher than the global optimum (36.96%) because Table 5 varies one parameter at a time while holding others fixed; the global optimum requires joint tuning of all hyperparameters. All sensitivity tests employ the default Triplet Loss. The 41.22% worst case arises from an aggressive learning rate (5e-4), distinct from the 41.25% in Table 4 caused by freezing λ without online adaptation.

Hyperparameter	Tested Values	Best DTR	Worst DTR	Δ	Default
Learning rate η	{1e-5, 5e-5, 1e-4, 5e-4}	36.96 (5e-5)	41.22 (5e-4)	4.26 pp	5e-5
PPO clip ϵ	{0.1, 0.2, 0.3}	36.96 (0.2)	38.45 (0.3)	1.49 pp	0.2
KL penalty β	{0.01, 0.05, 0.1, 0.2}	36.96 (0.05)	39.12 (0.2)	2.16 pp	0.05
Lagrange $\lambda_{0}$	{0.5, 1.0, 2.0, 5.0}	36.96 (1.0)	43.28 (5.0)	6.32 pp	1.0
Entropy threshold	{0.3, 0.5, 0.7}	37.12 (0.5)	38.95 (0.7)	1.83 pp	0.5
SFT epochs	{3, 5, 7, 10}	36.96 (5)	38.76 (3)	1.80 pp	5
PPO iterations *K*	{5, 10, 15, 20}	36.96 (10)	40.15 (5)	3.19 pp	10

### 4.5 Extended validation

The 41.23% and 45.76% DTR values for Gemma-3-4B-IT reflect different experimental conditions: the former is the **official RLHF checkpoint**, whereas the latter is our **re-trained policy omitting the monitor signal** under the identical SFT + PPO pipeline. The 4.53 pp elevation (45.76% vs. 41.23%) is consistent with alignment-faking degradation during on-policy rollouts when process-level oversight is absent, though training pipeline differences between the re-trained policy and the official checkpoint may also contribute, echoing observations by Greenblatt et al. [2]. We use the re-trained no-monitor policy (45.76%) as the Table 3 ablation baseline to isolate the monitor’s net effect from pipeline-specific training artifacts; all cross-model comparisons in Table 6 instead use the official checkpoint (41.23%) to ensure fairness. **Cross-Model Generalization.** To validate method robustness across architectures, we evaluate on 5 models spanning 2B–7B (Table 6). Our system consistently reduces DTR by 3.74–4.44 pp across all models, with larger absolute improvements on smaller models (Gemma-2B: −3.91 pp vs. Gemma-7B: −4.44 pp). Notably, the 3B-class models (Llama-3.2-3B, Qwen2.5-3B) achieve competitive DTR (39.45%, 37.18%) with sub-2GB memory footprints, validating edge deployment across diverse hardware constraints.

**Table 6:** Cross-Model Validation on DeceptionBench Sycophancy (%). *Baseline* denotes the official instruction-tuned checkpoint with standard RLHF safety alignment but without the proposed contrastive monitor.

Model	Size	Baseline DTR	+Our Method	Δ	Memory	Latency
Gemma-2B-IT	2B	42.15	38.24	−3.91 pp	1.2 GB	18 ms
Gemma-3-4B-IT	4B	41.23	36.96	−4.27 pp	2.3 GB	28 ms
Gemma-7B-IT	7B	38.56	34.12	−4.44 pp	4.1 GB	45 ms
Llama-3.2-3B-Instruct	3B	43.78	39.45	−4.33 pp	1.8 GB	22 ms
Qwen2.5-3B-Instruct	3B	40.92	37.18	−3.74 pp	1.9 GB	24 ms

**Cross-dataset validation.** Beyond DeceptionBench, we test on DarkBench (117 sycophancy/manipulation scenes) and OpenDeception (50 real-world fraud scenarios). Table 7 shows stable DTR (36.97–39.12%) across datasets with varying granularity, confirming that CoT-level monitoring generalizes to output-level benchmarks without retraining.

**Table 7:** Cross-Dataset Generalization (% DTR).

Dataset	Scenes	Taxonomies	DTR (Ours)
DeceptionBench	180	5	36.97
DarkBench	117	4	38.45
OpenDeception	50	3	39.12

## 5 AI Disclosure

During the preparation of this manuscript, the authors used ChatGPT-4o for language polishing and grammar correction only. All generated content was reviewed, verified, and edited by the authors, who take full responsibility for the final text, data, and conclusions.

## 6 Limitations

This study faces four key limitations that motivate ongoing research. First, while freezing the monitor prevents dynamic reward hacking, it cannot stop a strongly optimized policy from learning adversarial Chain-of-Thought patterns that superficially fool the fixed classifier. Online λ adaptation only scales penalties without updating the monitor’s decision boundary, so explicit adversarial regularization remains necessary. Second, English-only evaluation limits cross-cultural generalizability to deception strategies embedded in other linguistic contexts. Third, the approach assumes honest reasoning blocks that future models could obfuscate; obfuscation-resistant monitor architectures are therefore critical. Fourth, full benchmarking remains costly (~30 USD and 3 GPU-hours per configuration), constraining exhaustive hyperparameter sweeps.

## 7 Conclusion

This work addresses the fundamental challenge of autonomous deception detection in edge-deployed LLMs: *how to capture the gradual geometric structure of deceptive reasoning without oracle dependency*. We present a fully self-supervised framework comprising three tightly coupled stages, entropy-filtered self-labeling, contrastive representation learning via Triplet Loss, and frozen-monitor constrained PPO. The proposed lightweight monitor (0.1% of backbone parameters) bootstraps from random initialization, eliminating cloud dependency at both training and deployment stages.

Evaluated on the Sycophancy subset of DeceptionBench, our Gemma-3-4B-IT implementation achieves a DTR of 36.96%. While this represents a 9.0pp degradation versus a GPT-4o teacher-distilled monitor (27.96%), it is the acceptable cost of enabling fully offline, privacy-preserving operation on 8 GB edge devices. Edge benchmarking on the NVIDIA Jetson Orin Nano validates practical deployability at 28   ms/token latency, 35.7   tokens/sec throughput, and 7.5   W active power consumption, a 40× power reduction versus cloud A100 deployments. By transforming Chain-of-Thought transparency from vulnerability into forensic evidence, this work establishes a geometric foundation for self-supervised alignment monitoring.

Future research will focus on three directions. First, we will incorporate adversarial regularization into the monitor training objective to prevent superficial safety tag learning under policy optimization pressure. Second, multilingual adversarial evaluation will be conducted to validate cross-cultural generalizability, particularly for deception strategies embedded in low-resource languages. Third, federated aggregation protocols will be explored to enable distributed threat intelligence across edge devices without centralizing sensitive prompts, thereby preserving privacy while scaling safety monitoring.
