# Supplementary material for: Contrastive representation learning for self-supervised deception detection in edge LLMs
Source: PLoS One. 2026 Aug 14;21(8):e0354894. doi: 10.1371/journal.pone.0354894 (PMC13475878; doi:10.1371/journal.pone.0354894)
Supplement: Supplementary file 1 [file pone.0354894.s002.pdf]

Dear Academic Editor and Reviewers,

Thank you for the thorough and constructive feedback on our revised manuscript (PONE-D-26-11514R1). We have carefully addressed every comment raised by Reviewers #3 and #4. Below, we provide a point-by-point response detailing the changes made. All modifications are highlighted in the revised manuscript using tracked changes (marked-up version) and are also present in the clean version.

Reviewer #3 Point 1: Discussion of Costa et al. (2025) and comparison with manifold-aware scoring

Section 2.5；
"Recent work by Costa et al. [28] similarly employs manifold-aware ..."

Reviewer #3 Point 2: Missing core table of main detection results (7 baseline models)

New Section 4.3, Table 3；
We apologize for this omission. We have inserted a new Table 3 in Section 4.3 'Detection Efficacy on DeceptionBench'. This table reports DTR, F1, Precision, and Recall on the full-set DeceptionBench (180 adversarial scenes, 5 deception taxonomies) for all seven baseline models and our contrastive-monitoring variant (36.97% DTR). Note: this full-set result (36.97%) is distinct from the Sycophancy-subset results (36.96%) reported in Tables 4 – 6 and Fig 4.

Reviewer #3 Point 3: Inconsistency between Fig 2 caption and figure content

Thank you for catching this. We have completely rewritten the caption of Fig 2 to accurately reflect the Input-Generation-Filter-Iteration pipeline and the iterative refinement loop.

Reviewer #3 Point 4: Data inconsistency between Table 6 (Baseline 41.23%) and Table 4 (No monitor 45.76%)

The reviewer noted an apparent conflict, we apologize for the confusion caused by inconsistent terminology across tables. We have added a clarifying explanation in Section 4.5 and Table 4 caption that explicitly distinguishes the two conditions: the 41.23% baseline in Table 6 refers to the official RLHF checkpoint, whereas the 45.76% 'No monitor' in Table 4 refers to our re-trained policy under the identical SFT+PPO pipeline without the monitor signal.

Reviewer #4 Point 1: Citation of Rahman et al. (2024)

We have incorporated this reference as requested. Rahman et al. (2024) [29] is now cited in Section 2.4 ("Edge deployment of LLMs") to contextualize on-device deception monitoring in

clinical settings.

Additional response to Reviewer #4's Q2 (Technical soundness) and Q4 (Data availability)

Reviewer #4's "No" responses to Q2 and Q4 suggest potential concerns about experimental transparency and data completeness. To proactively address these:

1. Technical soundness (Q2): We have strengthened the manuscript by adding the missing Table 3 (main detection results) and clarifying the baseline definitions in Table 4 caption and Section 4.5, ensuring that all experimental comparisons are fully transparent and reproducible.
2. Data availability (Q4): We have expanded the Data Availability Statement to explicitly confirm that all per-sample DTR scores, hidden-state vectors, and entropy traces underlying Figs 3 – 5 and Tables 3 – 7 are deposited in **S1_Data.xlsx** with clearly labeled sheets (e.g., "MainResults", "Ablation", "CrossModel", "CrossDataset"). The code and curated adversarial prompts remain publicly available at the Gitee repository previously disclosed.

Summary of Changes:

Reviewer	Comment	Action Taken	Location
R3-1	Costa et al. discussion	Added comparative paragraph	Section 2.5
R3-2	Missing main results table	Added Table 3 with 7-model DTR/F1/P/R on full-set DeceptionBench	Section 4.3
R3-3	Fig 2 caption mismatch	Rewrote caption + body text	Fig 2, page 6
R3-4	Table 6 vs Table 4 conflict	Added clarifying explanation in Section 4.5 and Table 4 caption	Section 4.5, Table 4 caption
R4	Rahman et al. citation	Added citation + contextual discussion	Section 2.4

We believe these revisions fully address all reviewer concerns and strengthen the manuscript's clarity, completeness, and reproducibility. We thank the reviewers for their valuable feedback, which has significantly improved the quality of the paper.

Sincerely,

Feng An (on behalf of all authors)
Corresponding Author
Department of Biotechnology
Suzhou Industrial Park Institute of Services Outsourcing
Suzhou, Jiangsu, China
Email: anf@siso.edu.cn
