# Supplementary material for: Contrastive representation learning for self-supervised deception detection in edge LLMs
Source: PLoS One. 2026 Aug 14;21(8):e0354894. doi: 10.1371/journal.pone.0354894 (PMC13475878; doi:10.1371/journal.pone.0354894)
Supplement: Supplementary file 2 [file pone.0354894.s001.doc]

**Reviewer 1 Comment Q1: The paper's innovativeness and research contributions are not clearly articulated... It is recommended that the authors clearly list the main contributions of this paper at the end of the introduction, explaining the differences from existing research point by point.**

We thank the reviewer for this critical feedback. We revision the ideas and focus on contrastive learning, replace the BCE loss. We changed the title, abstract, and full text to make the innovation clear and practical.

Change title to “Contrastive Representation Learning for...”;

Add limitations and contributions at the end of Introduction;

Rewrite Sec 3.2;

**Reviewer 1 Comment Q2: The introduction provides considerable background information... but the paper does not clearly propose a specific research question... The research objective is not focused enough.**

We appreciate the reviewer's emphasis on research focus, the reviewer is absolutely correct. We tried to make the research objective clear and focus, remove those backgrounds and integration of existing methods. We focus on contrastive learning in SFT as our innovation, and from the title to the full text, we changed to highlight the object. We have made the following structural changes to the Introduction:

Change title to “Contrastive Representation Learning for...”;

Added explicit Core Research Question in Introduction;

Added shortcomings of Existing Approaches in Introduction

And tightened contribution paragraph to explicitly state

**Reviewer 1 Comment Q3: The current experiments... are primarily validated on a single model... the overall experimental scale is still relatively small... It is recommended that the authors appropriately expand the experimental section.**

We thank the reviewer for this constructive criticism. We have substantially expanded the experimental section with three major additions:

Cross-model validation (**Table 5**), Cross-dataset generalization (**Table 6**)

**Reviewer 1 Comment Q4: The paper provides a relatively detailed process... but the explanation of "why this method is effective" is insufficient... more like an engineering implementation process, with relatively insufficient academic theoretical support.**

We thank the reviewer for this insightful critique. We have added theoretical justification at four key points in the Methods section:

Self-label reliability (Information Theory), end of Sec 3.1;

Limitation of Standard BCE Formulation, in Sec 3.2;

Why Contrastive Learning Outperforms BCE, end of Sec 3.2;

Frozen-monitor stability (Optimization Theory), Sec 3.3;

Iterative refinement convergence (Bootstrap Theory): Sec 3.4;

These additions transform the Methods section from procedural description into theoretically grounded design, explicitly connecting each engineering choice to its underlying mechanism.

**Reviewer 1 Comment Q5: The overall academic expression... still needs further standardization. Some sections focus excessively on background and application scenarios... the abstract and introduction contain extensive descriptions of "edge device deployment" and "offline operation," but the highlighting of methodological innovation and academic significance is not sufficiently evident.**

We thank the reviewer for this critical assessment of the paper's scholarly presentation. We removed those background introduction and the “edge device deployment”, highlighting the contrastive learning, made revisions to refocus from engineering description to theoretical contribution:

Abstract restructuring;

Introduction reorganization;

Conclusion expansion;

Terminology standardization:

**Reviewer 2 Comment Q1: The manuscript should clearly indicate the urgency of the research problem and clearly list the issues to be addressed.**

We appreciate the reviewer's emphasis on problem framing clarity. We have made the following additions to the Introduction:

Added urgency paragraph;

Added enumerated research questions;

Revised contribution paragraph.

These additions transform the Introduction from a general background into a sharply motivated research agenda with clear problem-solution alignment.

**Reviewer 2 Comment Q2: Related literature on large models should be more comprehensively included, such as Potential of large language models in blockchain-based supply chain finance[J]. Enterprise Information Systems, 2025: 2541199. and Self-Supervised Behavioral Risk Monitoring for Large Language Models in Edge Intelligence Environments. Journal of AI Analytics and Applications, 3(3), 1-18. etc.**

We thank the reviewer for these valuable references. We have comprehensively updated the Related Work section to incorporate both suggested papers and strengthen our literature coverage:

[19] Yang et al. "Potential of large language models in blockchain-based supply chain finance" (Enterprise Information Systems, 2025).

[20] Chen Y, Al-Najjar I. "Self-Supervised Behavioral Risk Monitoring for Large Language Models in Edge Intelligence Environments" (Journal of AI Analytics and Applications, 2025).

**Reviewer 2 Comment Q3: Although the author claims to run on Jetson Orin Nano, he does not provide key indicators such as actual inference latency, power consumption, and throughput.**

We thank the reviewer for this important observation. We have added a dedicated **Table 2** (Edge Deployment Performance Benchmarks) to comprehensively address this concern.

**Reviewer 2 Comment Q4: Supplement hyperparameter sensitivity analysis and its impact on DTR.**

We thank the reviewer for this important suggestion. We have added comprehensive hyperparameter sensitivity analysis to validate the robustness of our results:

Added **Table 4** (Hyperparameter Sensitivity Analysis)

**Reviewer 2 Comment Q5: Conclusion is too brief and should more comprehensively display the research content, research conclusions, research prospects, etc. of the manuscript.**

We thank the reviewer for this import suggestion. We have substantially expanded the Conclusion section. And we add a limitation section before Conclusion.
