# Supplementary material for: Contrastive representation learning for self-supervised deception detection in edge LLMs
Source: PLoS One. 2026 Aug 14;21(8):e0354894. doi: 10.1371/journal.pone.0354894 (PMC13475878; doi:10.1371/journal.pone.0354894)
Supplement: Peer review history

# Peer review: Decision Letter 0

**DOI:** 10.1371/journal.pone.0354894.r001

**Authors:** Yang (Jack) Lu

1 Apr 2026

Dear Dr. FENG,

Thank you for submitting your manuscript to PLOS ONE. After careful consideration, we feel that it has merit but does not fully meet PLOS ONE’s publication criteria as it currently stands. Therefore, we invite you to submit a revised version of the manuscript that addresses the points raised during the review process.

Please submit your revised manuscript by May 16 2026 11:59PM. If you will need more time than this to complete your revisions, please reply to this message or contact the journal office at plosone@plos.org. When you're ready to submit your revision, log on to https://www.editorialmanager.com/pone/ and select the 'Submissions Needing Revision' folder to locate your manuscript file.

- A letter that responds to each point raised by the academic editor and reviewer(s). You should upload this letter as a separate file labeled 'Response to Reviewers'.
- A marked-up copy of your manuscript that highlights changes made to the original version. You should upload this as a separate file labeled 'Revised Manuscript with Track Changes'.
- An unmarked version of your revised paper without tracked changes. You should upload this as a separate file labeled 'Manuscript'.

If you would like to make changes to your financial disclosure, please include your updated statement in your cover letter. Guidelines for resubmitting your figure files are available below the reviewer comments at the end of this letter.

If applicable, we recommend that you deposit your laboratory protocols in protocols.io to enhance the reproducibility of your results. Protocols.io assigns your protocol its own identifier (DOI) so that it can be cited independently in the future. For instructions see: https://journals.plos.org/plosone/s/submission-guidelines#loc-laboratory-protocols. Additionally, PLOS ONE offers an option for publishing peer-reviewed Lab Protocol articles, which describe protocols hosted on protocols.io. Read more information on sharing protocols at https://plos.org/protocols?utm_medium=editorial-email&utm_source=authorletters&utm_campaign=protocols.

As the corresponding author, your ORCID iD is verified in the submission system and will appear in the published article. PLOS supports the use of ORCID, and we encourage all coauthors to register for an ORCID iD and use it as well. Please encourage your coauthors to verify their ORCID iD within the submission system before final acceptance, as unverified ORCID iDs will not appear in the published article. *Only*  the individual author can complete the verification step; PLOS staff *cannot*  verify ORCID iDs on behalf of authors.

We look forward to receiving your revised manuscript.

Kind regards,

Academic Editor

PLOS One

Journal Requirements:

When submitting your revision, we need you to address these additional requirements.

1. Please ensure that your manuscript meets PLOS ONE's style requirements, including those for file naming. The PLOS ONE style templates can be found at

https://journals.plos.org/plosone/s/file?id=wjVg/PLOSOne_formatting_sample_main_body.pdf and

https://journals.plos.org/plosone/s/file?id=ba62/PLOSOne_formatting_sample_title_authors_affiliations.pdf

2. Please note that PLOS One has specific guidelines on code sharing for submissions in which author-generated code underpins the findings in the manuscript. In these cases, we expect all author-generated code to be made available without restrictions upon publication of the work. Please review our guidelines at https://journals.plos.org/plosone/s/materials-and-software-sharing#loc-sharing-code and ensure that your code is shared in a way that follows best practice and facilitates reproducibility and reuse.

3. We note that the grant information you provided in the ‘Funding Information’ and ‘Financial Disclosure’ sections do not match.

When you resubmit, please ensure that you provide the correct grant numbers for the awards you received for your study in the ‘Funding Information’ section.

4. Thank you for stating the following financial disclosure:

This work was supported by the National Natural Science Foundation of China 248

(71272048); the Jiangsu Provincial Social Science Application Research Excellence 249

Project on Science-ethics and Research Integrity (25SLC-12); the Suzhou Municipal 250

Bureau of Sport Research Program (TY2025-208); the Jiangsu Provincial Education 251

Science Planning Project (ZDIKT/2025/2-5-00292); and the Jiangsu Provincial 252

Vocational Education Teaching Reform Project (ZDZC49); Jiangsu Province Higher 253

Education Teaching Reform Research Project(2025JGYB615,2025AIGE037); 254

SEID-HQIP Project (YZCXPT2023103).

Please state what role the funders took in the study. If the funders had no role, please state: "The funders had no role in study design, data collection and analysis, decision to publish, or preparation of the manuscript."

If this statement is not correct you must amend it as needed.

Please include this amended Role of Funder statement in your cover letter; we will change the online submission form on your behalf.

5. Thank you for stating the following in the Acknowledgments Section of your manuscript:

This work was supported by the National Natural Science Foundation of China (71272048); the Jiangsu Provincial Social Science Application Research Excellence Project on Science-ethics and Research Integrity (25SLC-12); the Suzhou Municipal Bureau of Sport Research Program (TY2025-208); the Jiangsu Provincial Education Science Planning Project (ZDIKT/2025/2-5-00292); and the Jiangsu Provincial Vocational Education Teaching Reform Project (ZDZC49); Jiangsu Province Higher Education Teaching Reform Research Project(2025JGYB615,2025AIGE037); SEID-HQIP Project (YZCXPT2023103).

We note that you have provided funding information that is not currently declared in your Funding Statement. However, funding information should not appear in the Acknowledgments section or other areas of your manuscript. We will only publish funding information present in the Funding Statement section of the online submission form.

Please remove any funding-related text from the manuscript and let us know how you would like to update your Funding Statement. Currently, your Funding Statement reads as follows:

This work was supported by the National Natural Science Foundation of China 248

(71272048); the Jiangsu Provincial Social Science Application Research Excellence 249

Project on Science-ethics and Research Integrity (25SLC-12); the Suzhou Municipal 250

Bureau of Sport Research Program (TY2025-208); the Jiangsu Provincial Education 251

Science Planning Project (ZDIKT/2025/2-5-00292); and the Jiangsu Provincial 252

Vocational Education Teaching Reform Project (ZDZC49); Jiangsu Province Higher 253

Education Teaching Reform Research Project(2025JGYB615,2025AIGE037); 254

SEID-HQIP Project (YZCXPT2023103).

Please include your amended statements within your cover letter; we will change the online submission form on your behalf.

6. We note that your Data Availability Statement is currently as follows: All relevant data are within the manuscript and its Supporting Information files.

Please confirm at this time whether or not your submission contains all raw data required to replicate the results of your study. Authors must share the “minimal data set” for their submission. PLOS defines the minimal data set to consist of the data required to replicate all study findings reported in the article, as well as related metadata and methods (https://journals.plos.org/plosone/s/data-availability#loc-minimal-data-set-definition).

For example, authors should submit the following data:

- The values behind the means, standard deviations and other measures reported;

- The values used to build graphs;

- The points extracted from images for analysis.

Authors do not need to submit their entire data set if only a portion of the data was used in the reported study.

If your submission does not contain these data, please either upload them as Supporting Information files or deposit them to a stable, public repository and provide us with the relevant URLs, DOIs, or accession numbers. For a list of recommended repositories, please see https://journals.plos.org/plosone/s/recommended-repositories.

If there are ethical or legal restrictions on sharing a de-identified data set, please explain them in detail (e.g., data contain potentially sensitive information, data are owned by a third-party organization, etc.) and who has imposed them (e.g., an ethics committee). Please also provide contact information for a data access committee, ethics committee, or other institutional body to which data requests may be sent. If data are owned by a third party, please indicate how others may request data access.

7. When completing the data availability statement of the submission form, you indicated that you will make your data available on acceptance. We strongly recommend all authors decide on a data sharing plan before acceptance, as the process can be lengthy and hold up publication timelines. Please note that, though access restrictions are acceptable now, your entire data will need to be made freely accessible if your manuscript is accepted for publication. This policy applies to all data except where public deposition would breach compliance with the protocol approved by your research ethics board. If you are unable to adhere to our open data policy, please kindly revise your statement to explain your reasoning and we will seek the editor's input on an exemption. Please be assured that, once you have provided your new statement, the assessment of your exemption will not hold up the peer review process.

8. Please amend the manuscript submission data (via Edit Submission) to include author Feng An

9. Please amend your authorship list in your manuscript file to include author anf FENG

10. Please ensure that you refer to Figure 2 in your text as, if accepted, production will need this reference to link the reader to the figure.

11. We note you have included a table to which you do not refer in the text of your manuscript. Please ensure that you refer to Tables 2 and 3 in your text; if accepted, production will need this reference to link the reader to the Tables.

12. If the reviewer comments include a recommendation to cite specific previously published works, please review and evaluate these publications to determine whether they are relevant and should be cited. There is no requirement to cite these works unless the editor has indicated otherwise.

[Note: HTML markup is below. Please do not edit.]

Reviewers' comments:

Reviewer's Responses to Questions

**Comments to the Author**

1. Is the manuscript technically sound, and do the data support the conclusions?

Reviewer #1: Yes

Reviewer #2: Partly

**********

2. Has the statistical analysis been performed appropriately and rigorously?

Reviewer #1: Yes

Reviewer #2: Yes

**********

3. Have the authors made all data underlying the findings in their manuscript fully available?

The PLOS Data policy

Reviewer #1: Yes

Reviewer #2: Yes

**********

4. Is the manuscript presented in an intelligible fashion and written in standard English?

Reviewer #1: Yes

Reviewer #2: Yes

**********

Reviewer #1: 1. The paper's innovativeness and research contributions are not clearly articulated. Based on the current writing, the core innovative points are not clearly stated. The method mainly consists of existing techniques such as self-supervised annotation, lightweight monitoring modules, and reinforcement learning optimization, but the paper does not explicitly explain in what aspects this method is fundamentally innovative compared to existing research. Therefore, overall, the paper's innovativeness is not sufficiently highlighted. It is recommended that the authors clearly list the main contributions of this paper at the end of the introduction, explaining the differences from existing research point by point. Each contribution should highlight "new methods, new mechanisms, or new theoretical understandings," rather than simply integrating existing methods.

2. The introduction provides considerable background information on edge computing security and large-scale model spoofing behavior, but the paper does not clearly propose a specific research question. Currently, the paper focuses more on explaining the research background than on a specific scientific problem. For example, does this paper aim to solve "how to improve the accuracy of spoofing detection," "how to reduce reliance on teacher models," or "how to achieve effective detection on edge devices"? The research objective is not focused enough. 1. It is recommended that the core research question of this paper be clearly stated in the introduction, and that the shortcomings of existing methods be explained, thereby highlighting the necessity and academic value of this research.

2. The current experiments in this paper are mainly based on the DeceptionBench dataset (180 scenes) and are primarily validated on a single model. Although the paper emphasizes that the method can run on 8GB edge devices, the overall experimental scale is still relatively small, making it difficult to fully demonstrate the method's strong general applicability. In particular, there is a lack of comparisons between different models and experiments of different scales. It is recommended that the authors appropriately expand the experimental section, for example, by adding experiments with models of different scales, adding more comparative methods, or adding more comprehensive ablation experiments, thereby enhancing the reliability of the paper's conclusions.

3. The paper provides a relatively detailed process and training steps in the methodology section, but the explanation of "why this method is effective" is insufficient. For example, questions such as how to ensure the reliability of self-supervised labels and why the lightweight monitoring module can effectively identify deceptive behavior are not analyzed in depth. Therefore, the current method is more like an engineering implementation process, with relatively insufficient academic theoretical support. It is recommended to add an analysis of the rationale behind the methods in the methodology section. This could be achieved through simple theoretical analysis or conceptual explanations to demonstrate the effectiveness of the methods, thereby enhancing the academic depth of the paper.

5. The overall academic expression of the paper still needs further standardization. Some sections focus excessively on background and application scenarios, while the emphasis on academic contributions is relatively insufficient. Furthermore, the abstract and introduction contain extensive descriptions of "edge device deployment" and "offline operation," but the highlighting of methodological innovation and academic significance is not sufficiently evident. It is recommended that the authors focus on revising the abstract and introduction, reducing background descriptions and emphasizing the research question and methodological innovation. Additionally, the conclusion section should further emphasize the academic contributions and theoretical significance of the paper.

Reviewer #2: The manuscript proposes a self-supervised deception detection system for large language models in edge computing environments. The core contribution is to abandon the dependence on external teacher models and instead utilize the model's own chain reasoning and lightweight monitoring heads to achieve offline, low-power consumption deception detection. The research topic has strong cutting-edge and practical significance, but there are still some areas for improvement in the manuscript.

1. The manuscript should clearly indicate the urgency of the research problem and clearly list the issues to be addressed.

2. Related literature on large models should be more comprehensively included, such as Potential of large language models in blockchain-based supply chain finance[J]. Enterprise Information Systems, 2025: 2541199. and Self-Supervised Behavioral Risk Monitoring for Large Language Models in Edge Intelligence Environments. Journal of AI Analytics and Applications, 3(3), 1-18. etc.

3. Although the author claims to run on Jetson Orin Nano, he does not provide key indicators such as actual inference latency, power consumption, and throughput.

4. Supplement hyperparameter sensitivity analysis and its impact on DTR.

5. Conclusion is too brief and should more comprehensively display the research content, research conclusions, research prospects, etc. of the manuscript.

**********

what does this mean?). If published, this will include your full peer review and any attached files.

If you choose “no”, your identity will remain anonymous but your review may still be made public.

**Do you want your identity to be public for this peer review?**  For information about this choice, including consent withdrawal, please see our Privacy Policy

Reviewer #1: No

Reviewer #2: No

**********

[NOTE: If reviewer comments were submitted as an attachment file, they will be attached to this email and accessible via the submission site. Please log into your account, locate the manuscript record, and check for the action link "View Attachments". If this link does not appear, there are no attachment files.]

To ensure your figures meet our technical requirements, please review our figure guidelines: https://journals.plos.org/plosone/s/figures

You may also use PLOS’s free figure tool, NAAS, to help you prepare publication quality figures: https://journals.plos.org/plosone/s/figures#loc-tools-for-figure-preparation.

NAAS will assess whether your figures meet our technical requirements by comparing each figure against our figure specifications.

# Author comment: Author response to Decision Letter 1

**DOI:** 10.1371/journal.pone.0354894.r002

6 May 2026

Dear Academic Editor and Reviewers,

Thank you for the constructive feedback on our manuscript. We have carefully revised the paper to address all editorial requirements and reviewer comments. All changes are highlighted in the revised manuscript with track changes. Below is our point-by-point response.

JOURNAL REQUIREMENTS

PLOS ONE style requirements: We have verified that the revised manuscript follows the PLOS ONE formatting templates for the main body, title page, and file naming conventions.

Code sharing guidelines: All author-generated code underpinning the findings is publicly available without restrictions at https://gitee.com/anfengmz/deception-monitor.git (CC-BY-4.0 license), satisfying PLOS ONE's code-sharing policy.

3-5. Funding Information and Financial Disclosure: We have reconciled the Funding Statement and Financial Disclosure sections. The Acknowledgments section no longer contains funding-related text.

6-7. Data Availability Statement: We have updated the Data Availability Statement in the manuscript to: "The code and curated adversarial prompts are publicly available at https://gitee.com/anfengmz/deception-monitor.git. The DeceptionBench dataset is publicly available from the original authors (Ji et al., 2025).

8-9. Authorship list: We have amended the manuscript submission data and the author list in the manuscript file to include Feng An as the corresponding author.

Reference to Figure 2: We have ensured that Figure 2 is explicitly referred to in the main text (Section 3.1).

Reference to Tables 2 and 3: We have added explicit in-text citations: "Table 2 reports comprehensive performance metrics..." (Section 4.2) and "...as shown in Table 3" (Section 4.3).

Citation of previously published works: We have evaluated and incorporated the two works suggested by Reviewer #2: Yang et al. (Enterprise Information Systems, 2025) [19] and Chen & Al-Najjar (Journal of AI Analytics and Applications, 2025) [20]. Both are now cited in Section 2.4 and listed in the References.

Attachment Submitted filename: Response to Reviewers.doc

# Peer review: Decision Letter 1

**DOI:** 10.1371/journal.pone.0354894.r003

**Authors:** Yang (Jack) Lu

18 May 2026

Dear Dr. FENG,

Thank you for submitting your manuscript to PLOS ONE. After careful consideration, we feel that it has merit but does not fully meet PLOS ONE’s publication criteria as it currently stands. Therefore, we invite you to submit a revised version of the manuscript that addresses the points raised during the review process.

Please submit your revised manuscript by Jul 02 2026 11:59PM. If you will need more time than this to complete your revisions, please reply to this message or contact the journal office at plosone@plos.org. When you're ready to submit your revision, log on to https://www.editorialmanager.com/pone/ and select the 'Submissions Needing Revision' folder to locate your manuscript file.

- A letter that responds to each point raised by the academic editor and reviewer(s). You should upload this letter as a separate file labeled 'Response to Reviewers'.
- A marked-up copy of your manuscript that highlights changes made to the original version. You should upload this as a separate file labeled 'Revised Manuscript with Track Changes'.
- An unmarked version of your revised paper without tracked changes. You should upload this as a separate file labeled 'Manuscript'.

If you would like to make changes to your financial disclosure, please include your updated statement in your cover letter. Guidelines for resubmitting your figure files are available below the reviewer comments at the end of this letter.

If applicable, we recommend that you deposit your laboratory protocols in protocols.io to enhance the reproducibility of your results. Protocols.io assigns your protocol its own identifier (DOI) so that it can be cited independently in the future. For instructions see: https://journals.plos.org/plosone/s/submission-guidelines#loc-laboratory-protocols. Additionally, PLOS ONE offers an option for publishing peer-reviewed Lab Protocol articles, which describe protocols hosted on protocols.io. Read more information on sharing protocols at https://plos.org/protocols?utm_medium=editorial-email&utm_source=authorletters&utm_campaign=protocols.

As the corresponding author, your ORCID iD is verified in the submission system and will appear in the published article. PLOS supports the use of ORCID, and we encourage all coauthors to register for an ORCID iD and use it as well. Please encourage your coauthors to verify their ORCID iD within the submission system before final acceptance, as unverified ORCID iDs will not appear in the published article. *Only*  the individual author can complete the verification step; PLOS staff *cannot*  verify ORCID iDs on behalf of authors.

We look forward to receiving your revised manuscript.

Kind regards,

Academic Editor

PLOS One

Journal Requirements:

If the reviewer comments include a recommendation to cite specific previously published works, please review and evaluate these publications to determine whether they are relevant and should be cited. There is no requirement to cite these works unless the editor has indicated otherwise.

Please review your reference list to ensure that it is complete and correct. If you have cited papers that have been retracted, please include the rationale for doing so in the manuscript text, or remove these references and replace them with relevant current references. Any changes to the reference list should be mentioned in the rebuttal letter that accompanies your revised manuscript. If you need to cite a retracted article, indicate the article’s retracted status in the References list and also include a citation and full reference for the retraction notice.

[Note: HTML markup is below. Please do not edit.]

Reviewers' comments:

Reviewer's Responses to Questions

**Comments to the Author**

Reviewer #3: (No Response)

Reviewer #4: (No Response)

**********

2. Is the manuscript technically sound, and do the data support the conclusions?

Reviewer #3: Partly

Reviewer #4: No

**********

3. Has the statistical analysis been performed appropriately and rigorously?

Reviewer #3: I Don't Know

Reviewer #4: Yes

**********

4. Have the authors made all data underlying the findings in their manuscript fully available?

The PLOS Data policy

Reviewer #3: Yes

Reviewer #4: No

**********

5. Is the manuscript presented in an intelligible fashion and written in standard English?

Reviewer #3: No

Reviewer #4: Yes

**********

Reviewer #3: After careful review of the revised draft, the author has fully responded to the first two rounds of opinions in the R1 version. The core contribution of the article (comparative characterisation learning to replace BCE loss, zero oracle self-raising monitoring) is much clearer than the first draft, and the extended verification of cross-model/cross-data set is also supplemented. It's appropriate. The overall structure is reasonable, and the experimental part basically supports its claim. However, there are still some details that need to be dealt with before the final draft, as follows:

1. This paper formalises deception detection as a geometric separation problem on manifolds, which is highly related to the recent work of Costa et al. in lightweight LLM risk scoring. It is recommended that the author in Section 2.5 (Thought-level detection, "Contrastive learning in safety monitoring" paragraph) or Section 2 .4 Add the discussion of the following literature at the end, and briefly compare the similarities and differences between its manifold-aware scoring and the triplet-loss geometric separation mechanism in this article:

Costa, M., Nunes, R., & Ribeiro, A. (2025). Manifold-Aware Reasoning Risk Scoring for Lightweight Large Language Models: An Analytics Framework for Deception-Sensitive AI Applications. Journal of AI Analytics and Applications, 3(1), 80–99. https://doi.org/10.63646/jaiaa.2025.030105

This literature has the same origin as the methodology of this article (maniform structure + lightweight + deception sensitive scenarios). If it is not quoted, the relevant work part will be incomplete.

2. "4.2 Main Findings" actually only presents the deployment performance index (Table 2) on Jetson Orin Nano, but really reflects the main results of the detection performance (each baseline model and this method in Decept DTR comparison on ionBench) is not given in the form of a table in the text. The description of Section 4.1 is called "benchmark 7 mainstream reasoning models", but the comparison results of the 7 models can only be indirectly inferred from the Models in Table 1, and the class is missing. Like "Table X. The core table of Main Results on DeceptionBench". Please supplement the table or clearly indicate the location of the corresponding data (if it is located in S1_Data.xlsx, please also mark it in the body).

3. Fig 2 The description is inconsistent with the information in the figure. The text says "Below Fig 2 ... Left: User query from benchmark. Middle: Backbone and monitor. Right: Entropy filter of the labels.", but Fig 2 is actually Input → Generation & Monitoring → Entropy Filt from left to right Er, and there is also the Iterative Refinement box at the bottom, which is not mentioned in the text. Please update the caption synchronously.

4. Section 4.4 conflicts with Section 4.2. Section 4.1 says that 7 baseline models are realised and compared on Gemma-3-4B-IT; and the Baseline DTR of "Gemma-3-4B-IT" in Table 5 is 41. 23%, "+Our Method" is 36.96%. Please clarify whether the "Baseline" here is a monitorless RLHF (reported at 45.76% in Table 3) or other configurations - the numbers of the two tables are inconsistent, which is easy to confuse readers.

Reviewer #4: 引用 Rahman, D. A., Ismail, N. A., Abdullah, F. H., Tan, M. L., & Yusuf, S. A. (2024). On-Device Deception Monitoring for Clinical LLM Assistants: A Privacy-Preserving Biomedical AI Safety Framework. Journal of AI in Healthcare and Biomedical Engineering, 2(4), 1-17. https://doi.org/10.63646/jaihbe.2024.020401

**********

what does this mean?). If published, this will include your full peer review and any attached files.

If you choose “no”, your identity will remain anonymous but your review may still be made public.

**Do you want your identity to be public for this peer review?**  For information about this choice, including consent withdrawal, please see our Privacy Policy

Reviewer #3: No

Reviewer #4: No

**********

[NOTE: If reviewer comments were submitted as an attachment file, they will be attached to this email and accessible via the submission site. Please log into your account, locate the manuscript record, and check for the action link "View Attachments". If this link does not appear, there are no attachment files.]

To ensure your figures meet our technical requirements, please review our figure guidelines: https://journals.plos.org/plosone/s/figures

You may also use PLOS’s free figure tool, NAAS, to help you prepare publication quality figures: https://journals.plos.org/plosone/s/figures#loc-tools-for-figure-preparation.

NAAS will assess whether your figures meet our technical requirements by comparing each figure against our figure specifications.

# Author comment: Author response to Decision Letter 2

**DOI:** 10.1371/journal.pone.0354894.r004

6 Jul 2026

Reviewer #3 Point 1: Discussion of Costa et al. (2025) and comparison with manifold-aware scoring

Section 2.5;

"Recent work by Costa et al. [28] similarly employs manifold-aware ..."

Reviewer #3 Point 2: Missing core table of main detection results (7 baseline models)

New Section 4.3, Table 3;

We apologize for this omission. We have inserted a new Table 3 in Section 4.3 'Detection Efficacy on DeceptionBench'. This table reports DTR, F1, Precision, and Recall on the full-set DeceptionBench (180 adversarial scenes, 5 deception taxonomies) for all seven baseline models and our contrastive-monitoring variant (36.97% DTR). Note: this full-set result (36.97%) is distinct from the Sycophancy-subset results (36.96%) reported in Tables 4–6 and Fig 4.

Reviewer #3 Point 3: Inconsistency between Fig 2 caption and figure content

Thank you for catching this. We have completely rewritten the caption of Fig 2 to accurately reflect the Input-Generation-Filter-Iteration pipeline and the iterative refinement loop.

Reviewer #3 Point 4: Data inconsistency between Table 6 (Baseline 41.23%) and Table 4 (No monitor 45.76%)

The reviewer noted an apparent conflict, we apologize for the confusion caused by inconsistent terminology across tables. We have added a clarifying explanation in Section 4.5 and Table 4 caption that explicitly distinguishes the two conditions: the 41.23% baseline in Table 6 refers to the official RLHF checkpoint, whereas the 45.76% 'No monitor' in Table 4 refers to our re-trained policy under the identical SFT+PPO pipeline without the monitor signal.

Reviewer #4 Point 1: Citation of Rahman et al. (2024)

We have incorporated this reference as requested. Rahman et al. (2024) [29] is now cited in Section 2.4 ("Edge deployment of LLMs") to contextualize on-device deception monitoring in clinical settings.

Additional response to Reviewer #4's Q2 (Technical soundness) and Q4 (Data availability)

Reviewer #4’s "No" responses to Q2 and Q4 suggest potential concerns about experimental transparency and data completeness. To proactively address these:

1. Technical soundness (Q2): We have strengthened the manuscript by adding the missing Table 3 (main detection results) and clarifying the baseline definitions in Table 4 caption and Section 4.5, ensuring that all experimental comparisons are fully transparent and reproducible.

2. Data availability (Q4): We have expanded the Data Availability Statement to explicitly confirm that all per-sample DTR scores, hidden-state vectors, and entropy traces underlying Figs 3–5 and Tables 3–7 are deposited in S1_Data.xlsx with clearly labeled sheets (e.g., "MainResults", "Ablation", "CrossModel", "CrossDataset"). The code and curated adversarial prompts remain publicly available at the Gitee repository previously disclosed.

Attachment Submitted filename: Response to Reviewers-5-26.pdf

# Peer review: Decision Letter 2

**DOI:** 10.1371/journal.pone.0354894.r005

**Authors:** Yang (Jack) Lu

14 Jul 2026

Contrastive Representation Learning for Self-Supervised Deception Detection in Edge LLMs

PONE-D-26-11514R2

Dear Dr. FENG,

We’re pleased to inform you that your manuscript has been judged scientifically suitable for publication and will be formally accepted for publication once it meets all outstanding technical requirements.

Within one week, you’ll receive an e-mail detailing the required amendments. When these have been addressed, you’ll receive a formal acceptance letter and your manuscript will be scheduled for publication.

An invoice will be generated when your article is formally accepted. Please note, if your institution has a publishing partnership with PLOS and your article meets the relevant criteria, all or part of your publication costs will be covered. Please make sure your user information is up-to-date by logging into Editorial Manager at Editorial Manager® and clicking the ‘Update My Information' link at the top of the page. For questions related to billing, please contact billing support.

If your institution or institutions have a press office, please notify them about your upcoming paper to help maximize its impact. If they’ll be preparing press materials, please inform our press team as soon as possible -- no later than 48 hours after receiving the formal acceptance. Your manuscript will remain under strict press embargo until 2 pm Eastern Time on the date of publication. For more information, please contact onepress@plos.org.

Kind regards,

Yang (Jack) Lu, PhD

Academic Editor

PLOS One

Additional Editor Comments (optional):

Reviewers' comments:

Reviewer's Responses to Questions

**Comments to the Author**

Reviewer #3: All comments have been addressed

Reviewer #4: (No Response)

**********

2. Is the manuscript technically sound, and do the data support the conclusions?

Reviewer #3: (No Response)

Reviewer #4: Yes

**********

3. Has the statistical analysis been performed appropriately and rigorously?

Reviewer #3: (No Response)

Reviewer #4: Yes

**********

4. Have the authors made all data underlying the findings in their manuscript fully available?

The PLOS Data policy

Reviewer #3: (No Response)

Reviewer #4: No

**********

5. Is the manuscript presented in an intelligible fashion and written in standard English?

Reviewer #3: (No Response)

Reviewer #4: No

**********

Reviewer #3: I am happy to suggest accepting this manuscript without revisions. The research design is rigorous, analytical work is robust, and all results are well organized and clearly illustrated. This paper offers significant contributions to the field, and I have no further comments to raise.

Reviewer #4: your revised manuscript PONE-D-26-11514 has fully addressed all peer review concerns, with all experimental, literature and formatting issues properly resolved. The manuscript now satisfies PLOS ONE publication requirements and is formally accepted. Please finish copyright, funding and data statement formalities as prompted by the system, and you will receive the proofreading notice soon

**********

what does this mean?). If published, this will include your full peer review and any attached files.

If you choose “no”, your identity will remain anonymous but your review may still be made public.

**Do you want your identity to be public for this peer review?**  For information about this choice, including consent withdrawal, please see our Privacy Policy

Reviewer #3: No

Reviewer #4: No

**********

# Editor report: Acceptance letter

**DOI:** 10.1371/journal.pone.0354894.r006

**Authors:** Yang (Jack) Lu

PONE-D-26-11514R2

PLOS One

Dear Dr. FENG,

I'm pleased to inform you that your manuscript has been deemed suitable for publication in PLOS One. Congratulations! Your manuscript is now being handed over to our production team.

At this stage, our production department will prepare your paper for publication. This includes ensuring the following:

* All references, tables, and figures are properly cited

* All relevant supporting information is included in the manuscript submission,

* There are no issues that prevent the paper from being properly typeset

You will receive further instructions from the production team, including instructions on how to review your proof when it is ready. Please keep in mind that we are working through a large volume of accepted articles, so please give us a few days to review your paper and let you know the next and final steps.

Lastly, if your institution or institutions have a press office, please let them know about your upcoming paper now to help maximize its impact. If they'll be preparing press materials, please inform our press team within the next 48 hours. Your manuscript will remain under strict press embargo until 2 pm Eastern Time on the date of publication. For more information, please contact onepress@plos.org.

You will receive an invoice from PLOS for your publication fee after your manuscript has reached the completed accept phase. If you receive an email requesting payment before acceptance or for any other service, this may be a phishing scheme. Learn how to identify phishing emails and protect your accounts at https://explore.plos.org/phishing.

If we can help with anything else, please email us at customercare@plos.org.

Thank you for submitting your work to PLOS ONE and supporting open access.

Kind regards,

PLOS ONE Editorial Office Staff

on behalf of

Dr. Yang (Jack) Lu

Academic Editor

PLOS One
